# Changes in symptom burden and quality of life among women with uterine fibroids receiving relugolix combination therapy: a plain language summary

**DOI:** 10.57264/cer-2023-0194

**Published:** 2024-06-27

**Authors:** Elizabeth A Stewart, Andrea S Lukes, Roberta Venturella, Yulan Li, Elke Hunsche, Rachel B Wagman, Ayman Al-Hendy

**Affiliations:** 1 Mayo Clinic & Mayo Clinic Alix School of Medicine, Rochester, MN, USA; 2 Carolina Woman's Research & Wellness Center, Durham, NC, USA; 3 Magna Graecia University of Catanzaro, Catanzaro, Italy; 4 Sumitomo Pharma America, Marlborough, MA, USA; 5 Sumitomo Pharma Switzerland GmbH, Basel, Switzerland; 6 University of Chicago, Chicago, IL, USA

**Keywords:** heavy menstrual bleeding, lay summary, plain language summary, quality of life, symptom burden, uterine fibroids, women's health

## Abstract

**What is this summary about?:**

This is a summary of findings from two research studies (known as clinical trials). The studies looked at how well a medicine called relugolix combination therapy worked in women with heavy menstrual bleeding (heavy bleeding during a period) with uterine fibroids (noncancerous or benign growths in the uterus). In this analysis of the studies, researchers looked at how patients self-reported their uterine fibroid symptoms before and after taking relugolix combination therapy. Researchers also looked at how patients self-reported the impact of uterine fibroids on their health-related quality of life before and after taking relugolix combination therapy.

**What were the results?:**

Women took either relugolix combination therapy or placebo (a pill that contains no medicine) by mouth once daily for 24 weeks. Women completed the Uterine Fibroid Symptom and Quality of Life questionnaire (where “quality of life” refers to the women's health-related quality of life related to uterine fibroids) before, during, and after treatment. The questionnaire let researchers see if the women felt that relugolix combination therapy decreased the burden of uterine fibroid symptoms and improved the women's health-related quality of life related to uterine fibroids. More women said that they felt less distress due to their uterine fibroid symptoms and that their health-related quality of life related to uterine fibroids was better after taking relugolix combination therapy compared with women who took placebo.

**What do the results mean?:**

Relugolix combination therapy may lessen distress associated with uterine fibroid symptoms and improve health-related quality of life related to uterine fibroids.

**This is an abstract of the Plain Language Summary of Publication article.**

To read the full Plain Language Summary of this article, click here to view the PDF.

Link to original article here

